# Solubility of Carbon Dioxide in Secondary Butyl Alcohol at High Pressures: Experimental and Modeling with CPA

**DOI:** 10.1007/s10953-015-0361-x

**Published:** 2015-08-07

**Authors:** Sona Raeissi, Reza Haghbakhsh, Louw J. Florusse, Cor J. Peters

**Affiliations:** School of Chemical and Petroleum Engineering, Shiraz University, Mollasadra Ave., 71345 Shiraz, Iran; Delft University of Technology, Julianalaan 136, 2628 BL Delft, The Netherlands; Chemical Engineering Department, The Petroleum Institute, P.O. Box 2533, Abu Dhabi, United Arab Emirates; Department of Chemical Engineering and Chemistry, Separation Technology Group, Eindhoven University of Technology, Den Dolech 2, 5612 AZ Eindhoven, The Netherlands

**Keywords:** CO_2_, 2-Butanol, 2-Butyl alcohol, Cubic plus association, Vapor–liquid equilibria, Phase behavior

## Abstract

Mixtures of carbon dioxide and secondary butyl alcohol at high pressures are interesting for a range of industrial applications. Therefore, it is important to have trustworthy experimental data on the high-pressure phase behavior of this mixture over a wide range of temperatures. In addition, an accurate thermodynamic model is necessary for the optimal design and operation of processes. In this study, bubble points of binary mixtures of CO_2_ + secondary butyl alcohol were measured using a synthetic method. Measurements covered a CO_2_ molar concentration range of (0.10–0.57) % and temperatures from (293 to 370) K, with pressures reaching up to 11 MPa. The experimental data were modelled by the cubic plus association (CPA) equation of state (EoS), as well as the more simple Soave–Redlich–Kwong (SRK) EoS. Predictive and correlative modes were considered for both models. In the predictive mode, the CPA performs better than the SRK because it also considers associations.

## Introduction

Vapor–liquid equilibrium (VLE) data and accurate thermodynamic models for mixtures are basic requirements for the design, simulation, operation, and optimization of industrial processes. Mixtures involving CO_2_ and alcohols are of interest for a range of industrial ventures. Many of the investigated supercritical CO_2_ extraction tasks can be enhanced by the addition of an alcohol to CO_2_, as co-solvent, to increase the limited power that CO_2_ has for dissolving polar components. Carbon dioxide-expanded liquids are tuneable solvents for conducting chemical reactions, separations, and materials processing [1]. Other examples include processes involving biomaterials, such as the extraction of colorants from *Beta vulgaris* and the cempasuchil flour [2] or the *Pseudomonas cepacia* lipase-catalysed enantioselective transesterification of vinyl acetate + secondary butyl alcohol to butyl acetate + acetaldehyde in near critical carbon dioxide [3]. Butyl alcohol is also commonly used in synthesising high-porosity materials via the sol–gel processes and in supercritical drying processes [4].

Because of the interest in the phase behavior of the components involved, the solubility of carbon dioxide in secondary butyl alcohol has been determined previously by other researchers [2–7]. However, some of these studies are limited to only one or two specific temperatures. Even in the case of those studies which do include several temperatures, there is discrepancy among the experimental data, particularly at higher pressures. Therefore, considering the interest in this system for a variety industrial developments, the need for further CO_2_ solubility measurements in secondary butyl alcohol using accurate PVT equipment still exists, in order to help enrich the currently incoherent and inconclusive data available for this system. The present study aims to investigate the solubility of carbon dioxide in secondary butyl alcohol from an experimental, as well as a modeling perspective, since in addition to experimental data, modeling of the system is also vital for investigations of the phase behavior. The Soave–Redlich–Kwong (SRK) and Peng–Robinson (PR) equations of state (EoS) are simple and popular models, which are widely used in different industries [8]. Secuianu et al. measured and modeled VLE data of the carbon dioxide + secondary butyl alcohol system with a general cubic equation of state (GEOS), the PR EoS, and the SRK EoS using classical van der Waals (conventional two-parameter) mixing rules [5]. The apparatus used in their work was based on the static analytical method with liquid and vapor phase sampling. The apparatus was designed to operate at pressures below 30 MPa with an accuracy of 0.5 % and temperatures between (273 and 353 K) with an accuracy of 0.1 K [5]. Elizalde-Solis and Galicia-Luna used a static-analytic method, with an Anton Paar U-tube densitometer coupled to the stainless steel cell, for the simultaneous determination of the saturated densities of the equilibrium vapor and liquid phases. The uncertainty of pressure, temperature, density, and liquid and vapor molar compositions are 0.008 MPa, 0.05 K, 0.17 kg·m^3^, 1 and 2 %, respectively [6]. They correlated their experimental vapor–liquid equilibrium data using the Peng–Robinson EoS coupled with the Wong–Sandler mixing rules and succeeded in obtaining good agreement [6].

Stevens et al. developed a new apparatus for the experimental determination of vapor–liquid equilibria in systems containing low-volatility compounds and near-critical carbon dioxide. Their apparatus was tested by measuring the VLE of the system CO_2_ + butyl alcohol at 313.2 and 333.2 K and at pressures up to 11 MPa. They claimed that the measured mole fractions of the low-volatility compound in carbon dioxide had an accuracy better than 3 % [3]. They also modelled the CO_2_ solubility isotherms in secondary butyl alcohol using the PRSV EoS with the Wong–Sandler mixing rules, as well as the LCVM model (*t*-mPR EoS with an adapted UNIFAC–GE model). They concluded that the PRSV–WS predicts the solubilities better than the LCVM model [3].

However, the non-ideal mixture of CO_2_ + secondary butyl alcohol has not yet been modelled with the more complicated EoS which are particularly developed for associating systems, for example, the cubic-plus-association (CPA) or the different versions of the statistical association fluid theory (SAFT) [9]. CPA is among the more complicated EoS, developed on the basis of Wertheim’s theory. It is a combination of the physical interaction terms of SRK with an extra term that takes associations into account. Therefore, it can extend the capabilities of the cubic EoS to accurately model polar/hydrogen-bonding compounds. For example, in the oil and gas industries, it has the potential to incorporate hydrocarbons, gases, water, alcohols and glycols [9–13].

In this study, the high pressure bubble point data of binary mixtures of carbon dioxide + secondary butyl alcohol are measured using an accurate phase equilibrium measuring technique, for a number of different temperatures and up to high pressures. The experimental data are then modelled using the CPA EoS, and compared to the SRK as a representative of the simple cubic EoS. Both models are investigated in the predictive and correlative modes.

## Experimental

The Cailletet apparatus, used to carry out the solubility measurements in this study, has been explained in detail previously [14, 15]. It operates based on the synthetic method of phase equilibrium measurements, in which fixed (and known) amounts of substances are sealed within a glass equilibrium cell, and phase changes are observed visually upon changes of pressure at fixed temperature (or changes of temperature at fixed pressure). In the case of gas solubility measurements (bubble points) each measured pair of equilibrium temperature and pressure, coupled with the known overall composition which is essentially the composition of the liquid phase as the last bubble of vapor “dissolves”, indicates one equilibrium data point. This procedure, repeated over a number of temperatures, provides a constant–composition solubility curve on pressure–temperature coordinates. To make a more complete picture, such curves are obtained for a number of cell fillings (isopleths), each with a different overall composition of the mixture under study.

The Cailletet equipment itself, is composed of a Pyrex equilibrium cell in the shape of a tube with one open end and one closed end. The experimental sample is contained in the closed end of the tube, and the open end is immersed in mercury, and securely placed inside an autoclave. In this way, liquid mercury acts as the intermediate sealing and pressure-transmitting fluid between the sample and the hydraulic oil within the pressure-generating system. Pressure is generated on the system using a screw-type hand pump and it is measured using a dead–weight pressure gauge with an accuracy of 0.03 % of the reading. The Cailletet tube is fitted within a glass thermostat jacket, which has the role of allowing the circulation of the temperature-regulating thermostat liquid around the tube. A thermostat bath regulates the temperature of the thermostat liquid within a constancy better than 0.01 K. A platinum resistance thermometer indicates the temperature with a maximum error of 0.02 K. The accuracy of composition is within 0.001 in mole fraction.

Table 1 presents the purity and suppliers of the compounds experimentally investigated in this study.

**Table 1:** Purity, supplier and purification process of the compounds

Compound	Purity (%)	Supplier	Purification
Secondary butyl alcohol	≥99.5	Merck	No further purification
Carbon dioxide	≥99.95	Air Products	No further purification

## Modelling

The compressibility factor (*z*) of the cubic-plus-association EoS is actually the sum of the physical terms of the SRK EoS, and an association term as follows [16–23]:

1 $$ z = z^{{{\text{phys}} .}} + z^{{{\text{assoc}} .}} = \frac{1}{1 - b\rho } - \frac{a\rho }{RT(1 + b\rho )} - \frac{1}{2}\left( {1 + \rho \frac{\partial \ln g}{\partial \rho }} \right)\sum\limits_{i} {x_{i} } \sum\limits_{{A_{i} }} {(1 - X_{{A_{i} }} )} $$

where *a* and *b* are the pure energy parameter and co-volume parameter of SRK, respectively. The parameter of *a* is defined by the Soave-type temperature-dependent function of Eq. 2:

2 $$ a(T) = a_{0} \left[ {1 + c_{1} \left( {1 - \sqrt {T_{\text{r}} } } \right)} \right]^{2} $$

*ρ* and *x*_*i*_ are the density and mole fraction of component *i*, respectively. *X*_*Ai*_ is the mole fraction of the *A*-sites of component *i* which are not bonded to other molecules’ sites. *X*_*Ai*_ is a function of Δ_*AiBj*_, called the association strength which describes how strong the bond between site *A*, belonging to molecule *i*, and site *B* of molecule *j* is [9]. *X*_*Ai*_ is calculated by Eq. 3:

3 $$ X_{Ai} = \frac{1}{{1 + \rho \sum\limits_{j} {x_{j} \sum\limits_{{B_{j} }} {X_{{B_{j} }} \Delta^{AiBj} } } }} $$

4 $$ \Delta^{AiBj} = g(\rho )\left[ {\exp \left( {\frac{{\varepsilon^{AiBj} }}{RT}} \right) - 1} \right]b_{ij} \beta^{AiBj} $$

*g* is a simplified radial distribution function as proposed by Elliot et al. [24] and used by Kontogeorgis et al. [19]:

5 $$ g(\rho ) = \frac{1}{1 - 1.9\eta } $$

where:

6 $$ \eta = \frac{1}{4}b\rho $$

In this way, CPA uses three parameters, *a*_*0*_, *b*, and *c*_1_, in its physical part. Two further parameters, *β* and *ε*, which are the association volume and association energy, respectively, are used in the association term, but are only specified and used in the case of associating compounds. The five pure parameters can be estimated for each of the compounds in its pure state. This is done by simultaneous regression of liquid density and vapor pressure data [16], using Eq. 7 as the objective function of the optimization

7 $$ OF = \sum\limits_{i}^{Np} {\left( {\frac{{p_{i}^{{{ \exp }.}} - p_{i}^{{{\text{calc}}.}} }}{{p_{i}^{{{ \exp }.}} }}} \right)^{2} + \sum\limits_{i}^{Np} {\left( {\frac{{\rho_{i}^{{{ \exp }.}} - \rho_{i}^{{{\text{calc}}.}} }}{{\rho_{i}^{{{ \exp }.}} }}} \right)^{2} } } $$

where $p_{i}^{{{ \exp } .}}$ and $p_{i}^{{{\text{calc}} .}}$ are the experimental and the CPA calculated vapor pressures, respectively. It is necessary to specify an association scheme, i.e., the type and number of association sites for each species in order to determine the value of *X*_*Ai*_ for the associating components [9, 17]. The association scheme, proposed by Huang and Radosz [25], is adopted here. For alcohols, two association schemes are possible, namely 2*B* and 3*B*. Studies have shown that the use of the 3*B* scheme is more rigorous over the simpler 2*B* scheme; however the extra calculations do not lead to huge improvements over the simpler 2*B* [9, 17]. Therefore, the two-site association scheme (2*B*) is used here for secondary butyl alcohol.

In order to model the mixtures, the van der Waals one-fluid mixing rules (Eqs. 8–10) are used to calculate the energy and co-volume parameters, where *k*_*ij*_ and *l*_*ij*_ are the binary interaction parameters.

8 $$ a = \sum\limits_{i} {\sum\limits_{j} {x_{i} x_{j} a_{ij} } } $$

9 $$ a_{ij} = \sqrt {a_{i} a_{j} } (1 - k_{ij} ) $$

10 $$ \begin{aligned} b & = \sum\limits_{i} {\sum\limits_{j} {x_{i} x_{j} b_{ij} } } \\ b_{ij} & = \frac{{b_{i} + b_{j} }}{2}(1 - l_{ij} ) \\ \end{aligned} $$

In order to optimize the binary interaction parameter(s) of the mixtures, the following objective function is applied,

11 $$ OF = \sum\limits_{i}^{Np} {\left( {\frac{{p_{i}^{{{\text{calc}}.}} - p_{i}^{{{ \exp }.}} }}{{p_{i}^{{{ \exp }.}} }}} \right)^{2} } $$

## Results and Discussion

**Fig. 1:**
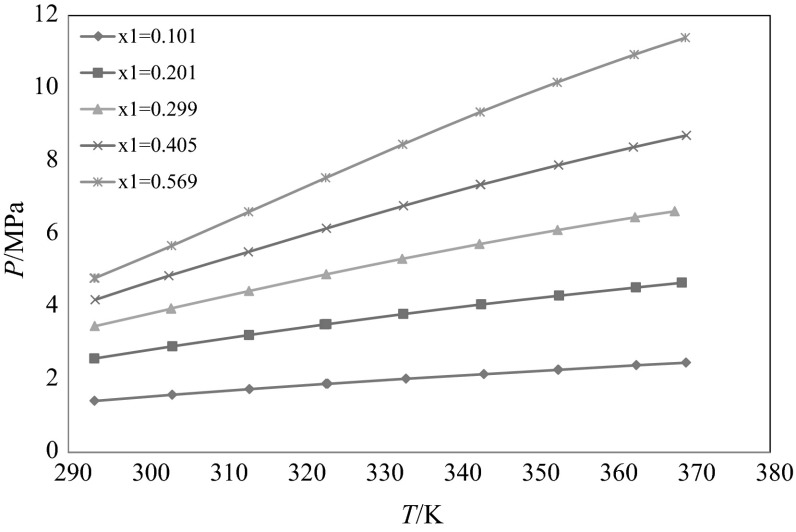
Experimentally measured bubble points pressure versus temperature for the CO_2_ (1) + secondary butyl alcohol (2) binary system at different molar concentrations

**Fig. 2:**
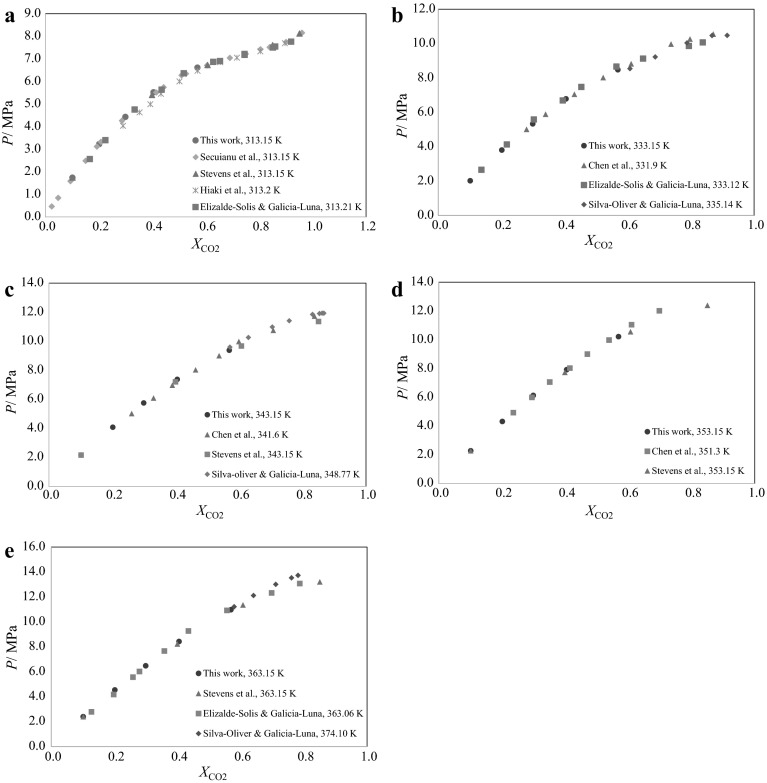
**a** Comparison of the solubility of CO_2_ (in mole fraction) in secondary butyl alcohol obtained in this work with literature data [3, 5–7] at 313 K; **b** comparison of the solubility of CO_2_ (in mole fraction) in secondary butyl alcohol obtained in this work with literature data [2, 4, 6] at 331–335 K; **c** comparison of the solubility of CO_2_ (in mole fraction) in secondary butyl alcohol obtained in this work with literature data [2–4] at 341–348 K; **d** comparison of the solubility of CO_2_ (in mole fraction) in secondary butyl alcohol obtained in this work with literature data [3, 4] at 351–353 K; **e** comparison of the solubility of CO_2_ (in mole fraction) in secondary butyl alcohol obtained in this work with literature data [2, 3, 6] at 363–374 K

Experimental bubble point pressures of binary mixtures of CO_2_ + secondary butyl alcohol were measured within a temperature range of (293–370) K for various molar concentrations of the mixture. Solubility pressures were determined up to pressures of about 114 bar. The experimental results of CO_2_ solubility are presented in Table 2 and Fig. 1. The solubility curves of Fig. 1 follow the general trend of CO_2_ solubility in liquids, where pressure increases with an increase of temperature. To compare the experimental data of this work with those measured previously in the literature, the *p*–*T* isopleths are interpolated into *p*–*x* isotherms and the results are presented in Table 3. Figure 2a–e compare the data graphically. This system is expected to have Type II phase behavior according to the classifications of van Konynenburg and Scott [26], although within the temperature range investigated in this study no three phase (LLV) equilibrium was observed. The changing slope at lower temperatures already hints towards a liquid–liquid split at lower temperatures.

**Table 2:** Experimental bubble point data for the system carbon dioxide (1) + secondary butyl alcohol (2)

*x*_1_	*T* (K)	*p* (MPa)	*T* (K)	*p* (MPa)	*T* (K)	*p* (MPa)
0.101	293.37	1.411	303.31	1.581	313.22	1.736
	323.05	1.881	323.26	1.881	333.31	2.021
	343.29	2.146	352.86	2.266	352.79	2.266
	362.83	2.391	369.20	2.461		
0.201	293.33	2.577	303.34	2.912	313.17	3.222
	322.86	3.512	323.10	3.517	332.95	3.797
	342.95	4.062	352.93	4.302	362.78	4.527
	368.67	4.657				
0.299	293.39	3.465	303.19	3.945	303.19	3.955
	313.16	4.430	323.09	4.890	332.82	5.315
	342.74	5.720	352.75	6.105	362.67	6.455
	367.76	6.620				
0.405	293.44	4.190	302.88	4.850	313.08	5.505
	323.1	6.150	332.98	6.770	342.84	7.355
	342.88	7.350	352.87	7.890	362.47	8.380
	369.27	8.705				
0.569	293.3	4.777	293.41	4.787	303.25	5.672
	313.11	6.602	322.99	7.537	332.87	8.457
	342.82	9.347	352.68	10.162	362.58	10.922
	369.13	11.387				

Standard uncertainties *u* are *u*(*T*) = 0.02 K, *u*(*x*_1_) = 0.001, and *u*(*p*) = 0.005 MPa

**Table 3:** Isothermal solubility data of liquid mole fraction *x*, temperature *T*, and pressure *p*, for carbon dioxide (1) + secondary butyl alcohol (2)

*x*_1_	0.101	0.201	0.299	0.405	0.569
*T* (K)	*p* (MPa)	*p* (MPa)	*p* (MPa)	*p* (MPa)	*p* (MPa)
293.15	1.407	2.571	3.451	4.175	4.761
303.15	1.578	2.905	3.949	4.854	5.671
313.15	1.735	3.222	4.430	5.518	6.607
323.15	1.881	3.520	4.890	6.161	7.550
333.15	2.017	3.801	5.327	6.778	8.477
343.15	2.147	4.064	5.738	7.363	9.370
353.15	2.270	4.309	6.120	7.911	10.208
363.15	2.391	4.537	6.470	8.416	10.970

Standard uncertainties *u* are *u*(*T*) = 0.02 K, *u*(*x*_1_) = 0.001, and *u*(*p*) = 0.005 MPa

Figure 2 also shows the previously published data on this binary mixture. It can be seen that there is some disagreement among the literature data from different laboratories. While the data of this work match rather well with those of Stevens et al. [3] and Secuianu et al. [5], the data by Hiaki et al. [7] are situated at lower pressures. At the higher temperatures, the curvatures of the solubility curves measured by Elizalde-Solis and Galicia-Luna [6] are greater than those of this work and also those by Secuianu et al. and Stevens et al., so their solubility pressures fall below ours at lower concentrations, while rising to pressures higher than the curves of this work at mid-range concentrations, and then again fall to lower pressures at high concentrations of CO_2_ (see for example the curves at 333 and 363 K). In contrast, the data by Chen et al. [4] (for example, see the isotherm at 351 K) are the opposite and have less curvature than those of Stevens et al. [3], Secuianu et al. [5], and this work. Although discrepancies are also observed among the various literature data available at all temperatures, the differences decrease at lower temperatures and pressures, such that the data at 313 K do not show alarming differences.

The *p*–*x* isotherms in Table 3 have been modeled with both the CPA and SRK EoS. The critical properties of secondary butyl alcohol and carbon dioxide, necessary in these models, are given in Table 4. Also in the case of CPA, in addition to the critical properties, the CPA parameters of the compounds should be calculated. However, since the pure component CPA parameters are already available in the literature for both secondary butyl alcohol and carbon dioxide, they are used directly as presented in Table 4. Carbon dioxide was assumed to be completely inert and its interactions with the secondary butyl alcohol molecules were limited to physical ones and so only the three physical parameters were attributed to CO_2_ with no consideration of association sites. However, as can been seen in Table 4, secondary butyl alcohol is given five pure CPA parameters including the physical and associating parameters, because it is an alcohol.

**Table 4:** Pure component critical parameters and CPA parameters for the experimented compounds

Compound	Pure CPA parameters^a^	Critical parameters [29]
Secondary butyl alcohol	*a*_0_ = 15.6063/bar·L^2^·mol^−2^	*T*_*c*_ = 536.05/K
	*b* = 0.0797/L·mol^−1^	*p*_*c*_ = 41.79/bar
	*c*_1_ = 0.9239	*ω* = 0.574
	*ε* = 210.00/bar·L·mol^−1^	
	*β* = 0.0041	
Carbon dioxide	*a*_0_ = 3.5/bar·L^2^·mol^−2^	*T*_*c*_ = 304.12/K
	*b* = 0.0272/L·mol^−1^	*p*_*c*_ = 73.74/bar
	*c*_1_ = 0.76	*ω* = 0.225

^a^Pure CPA parameters of secondary butyl alcohol and carbon dioxide were taken from Voutsas et al. [27] and Carvalho et al. [28], respectively

**Fig. 3:**
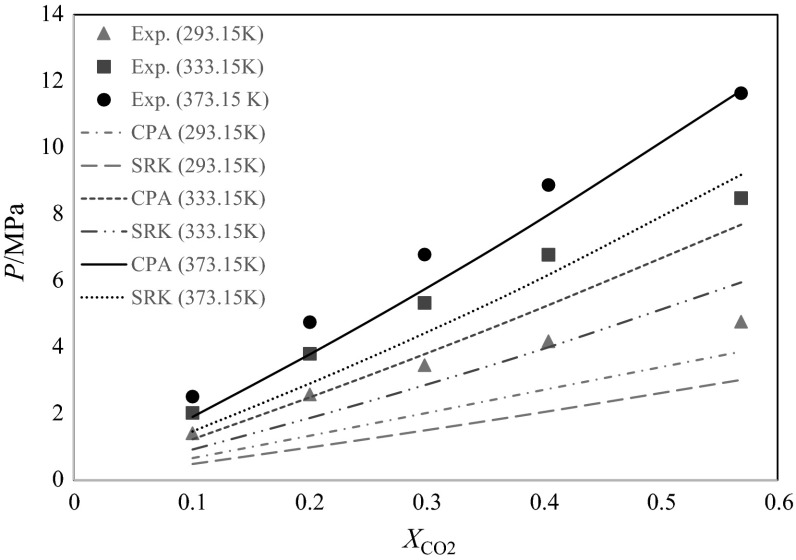
Comparison of CPA and SRK EoS predictions without the use of binary interaction coefficients at temperatures of 293.15, 333.15, and 373.15 K and different carbon dioxide mole fractions

The purely predictive results of the CPA and the SRK EoS, without using any binary interaction parameters (*k*_12_ and *l*_12_ are set to zero), are shown in Fig. 3. In this figure (and also the figures that follow), three sets of temperatures have been investigated as representatives of the experimental data, including the minimum, maximum and an interpolated middle temperature of the data (293.15, 333.15 and 373.15 K). The mixture of secondary butyl alcohol + carbon dioxide is non-ideal. This system’s non-ideality results from the presence of an associating compound (secondary butyl alcohol), having self-association between its molecules. In order to have a numerical comparison between the different models concerning their deviations from experimental data, the statistical parameter of percentage absolute average relative deviation (*AARD%*) has been used as follows

12 $$ AARD\% = \frac{1}{N}\sum\limits_{i}^{N} {\left| {\frac{{p_{i}^{{{\text{calc}}.}} - p_{i}^{{{ \exp }.}} }}{{p_{i}^{{{ \exp }.}} }}} \right|} \times 100, $$

where *p*^calc.^ and *p*^exp.^ are the EoS-predicted and the experimental bubble point pressures, respectively, and *N* is the total number of experimental data.

At 293.15 K, the CPA and SRK predicted the bubble point pressures of the mixture with an *AARD%* of 39.2 and 54.2 %, respectively. As the temperature increased, the *AARD%* decreased, respectively, to 26.8 and 44.5 % at 333.15 K, and 14.1 and 33.3 %, respectively, at the temperature of 373.15 K. The overall *AARD%* of the CPA and SRK for all of the isotherms between 293.15 and 373.15 K are 26.7 and 44.2 %, respectively. Therefore, without using any binary interaction parameters, both CPA and SRK give very poor results. However, the CPA predicts the bubble points of the system at different temperatures with much smaller deviations than the SRK.

To obtain better results, these equations were also investigated by optimizing binary interaction parameters in both of the models at different temperatures, by use of a genetic algorithm. For this purpose, *k*_12_ was considered as the fitting parameter and *l*_12_ was set to zero, using the objective function defined by Eq. 11. The optimized values of *k*_12_ for the CPA, as well as *k*_12_ for the SRK, are reported in Table 5.

**Table 5:** Optimized binary interaction parameters for the SRK and CPA EoS at different temperatures

*T* (K)	SRK EoS	CPA EoS
	*k*_12_	*k*_12_
293.15	0.144	0.103
303.15	0.141	0.098
313.15	0.138	0.093
323.15	0.134	0.085
333.15	0.132	0.080
343.15	0.124	0.073
353.15	0.126	0.065
363.15	0.115	0.060
373.15	0.105	0.040

**Fig. 4:**
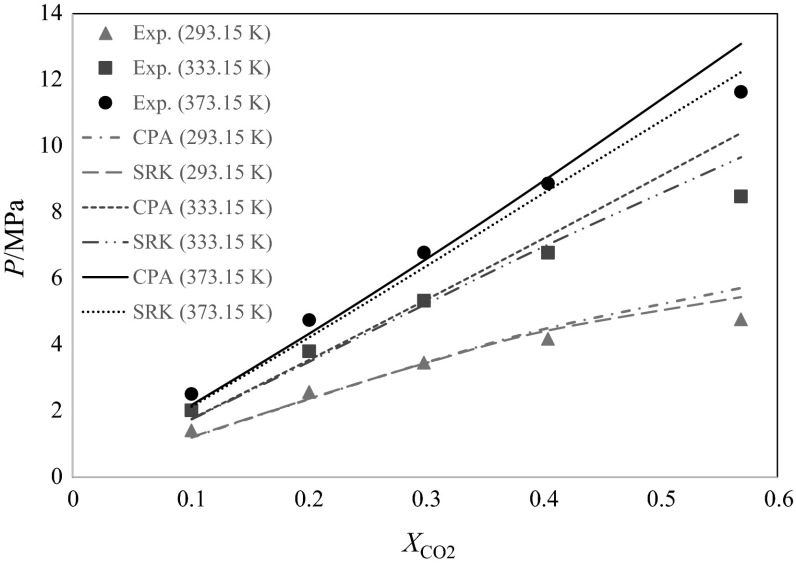
Comparison of correlative modes of CPA and SRK EoS to experimental data at different temperatures and carbon dioxide compositions. For SRK: *k*_12_ = 0.1436 at *T* = 293.15 K, *k*_12_ = 0.1315 at *T* = 333.15 K, *k*_12_ = 0.1053 at *T* = 373.15 K. For CPA: *k*_12_ = 0.1030 at *T* = 293.15 K, *k*_12_ = 0.0800 at *T* = 333.15 K, *k*_12_ = 0.0400 at *T* = 373.15 K

At the higher temperatures investigated, the systems tend to less nonideal behavior, so the values of the *k*_12_ fitting parameter decrease. Also as expected, the values of *k*_12_ of SRK are larger than those for the CPA, because SRK is a simple cubic EoS that considers only van der Waals type forces between its molecules, and is theoretically less suitable for this nonideal associating mixture than the CPA. Figure 4 shows the graphical comparison between SRK and CPA in the correlative mode at three different temperatures. Both the SRK and the CPA show acceptable agreement with the experimental data in the correlative mode and have similar results for the bubble points of the mixture, however, SRK is applying larger interaction parameters to achieve this. The values of *AARD%* of CPA and SRK are 10.2 and 8.5 %, respectively, at 293.15 K, 9.9 and 8.4 % at 333.15 K, and 7.9 and 8.0 % at 373.15 K, respectively.

It can be concluded that the use of adjustment parameters can greatly improve the SRK EoS, enabling it to approach within acceptable agreement with the experimental points. The overall *AARD%* of the CPA and the SRK for all of the data are 9.5 and 8.3 %, respectively. Although the error results are slightly better for the optimized SRK, it can still be argued that the CPA is the more suitable and theoretically sound model, due to the much smaller values of the binary interaction parameters used. This is because it takes into account the strong association interactions between the polar secondary butyl alcohol molecules, in addition to the physical interactions [9].

In order to compare the modeled phase behavior of carbon dioxide + secondary butyl alcohol with those in literature, Table 6 presents the *AARD%* values obtained in this study by the correlative SRK and CPA models and the three models of Secuianu et al. [5], PR, SRK and GEOS. However, Secuianu et al. used the classical van der Waals mixing rule by optimizing two binary interaction parameters (both *k*_*ij*_ and *l*_*ij*_). As can be seen from the table, they calculated very similar *AARD%* values for the PR and SRK as obtained by our model, even though they optimized two binary interaction coefficients, while in this work only one binary parameter (*k*_*ij*_) was fitted to the experimental data. The small SRK error differences between this work and that of Secuianu et al., in addition to the number of fitting parameters, is due to the differences in the pressure and composition range of the two studies and the number of points used to calculate *AARD%*. The GEOS model shows the best results, because in addition to binary interaction parameters, GEOS also incorporates pure parameters for the compounds which are optimized to the experimental data, and so, takes advantage of a larger number of fitting parameters.

**Table 6:** Comparison of percentage absolute average relative deviation (*AARD%*) in bubble point pressures for the carbon dioxide + secondary butyl alcohol system, calculated by different models

*T* (K)	*p* (MPa)	This study		*T* (*K*)	*p* (MPa)	Secuianu et al. [5]			Refs.
		SRK	CPA			GEOS	PR	SRK	
293.15	1.41–4.76	8.5	10.2	293.24	1.42–5.49	8.0	10.6	10.7	[3]
303.15	1.58–5.67	8.8	10.4	303.15	0.5–6.73	6.2	7.7	8.3	[5]
313.15	1.73–6.61	8.9	10.5	313.15	0.46–8.15	7.1	11.3	11.8	[5]
323.15	1.88–7.55	8.8	10.4	323.21	1.89–9.09	5.9	9.1	9.8	[3]
333.15	2.02–8.48	8.4	9.9	333.16	2.02–10.44	4.8	8.2	9.1	[3]
343.15	2.15–9.37	8.1	9.3	343.23	2.16–11.42	3.9	8.2	9.1	[3]
353.15	2.27–10.21	7.4	8.7	353.42	2.27–12.43	2.9	7.2	8.1	[3]
363.15	2.39–10.97	7.2	8.1	363.70	2.40–13.16	2.5	7.0	7.7	[3]
373.15	2.51–11.63	8.1	8.0	373.67	2.51–13.81	1.9	6.3	6.7	[3]

## Conclusions

Bubble point pressures were determined experimentally for binary mixtures of carbon dioxide and 2-butyl alcohol. The *p*–*T* curves obtained at each composition showed the typical positive slope encountered with CO_2_ solubility in liquids. Rather high pressures, up to 11 MPa, are required to dissolve carbon dioxide in secondary butyl alcohol in equimolar ratios, especially at higher temperatures. The measured data were compared to those in literature and the previously inconclusive differences among the data of various groups is now aided with a new dataset, which coincides well with two of the literature studies [3, 5], perhaps making the choice among the various datasets easier for the future.

Also the phase behavior of this system was modeled using the SRK EoS and the CPA EoS, both in predictive and correlative modes. The SRK EoS, with optimized binary parameters, correlates the experimental data with acceptable precision, with an *AARD%* value of 8.3 %. The optimized CPA EoS, can correlate the phase behavior of the system with an *AARD%* of 9.5 %, however, by using smaller binary interaction parameters with respect to the SRK.
